# Systematic characterization of germline variants from the DiscovEHR study endometrial carcinoma population

**DOI:** 10.1186/s12920-019-0504-9

**Published:** 2019-05-03

**Authors:** Jason E. Miller, Raghu P. Metpally, Thomas N. Person, Sarathbabu Krishnamurthy, Venkata Ramesh Dasari, Manu Shivakumar, Daniel R. Lavage, Adam M. Cook, David J. Carey, Marylyn D. Ritchie, Dokyoon Kim, Radhika Gogoi, on behalf of the DiscovEHR collaboration

**Affiliations:** 1 Department of Genetics, Institute for Biomedical Informatics, Perelman School of Medicine, University of Pennsylvania, Philadelphia, PA 19104 USA; 2 Biomedical & Translational Informatics Institute, Geisinger Health System, Danville, PA 17822 USA; 3 Weis Center for Research, Geisinger Medical Center, Danville, PA 17822 USA; 4 Huck Institute of the Life Sciences, Pennsylvania State University, University Park, PA 16802 USA; 5 Department of Biostatistics, Epidemiology and Informatics, Perelman School of Medicine, University of Pennsylvania, Philadelphia, USA; 6 Institute for Biomedical Informatics, University of Pennsylvania, Philadelphia, USA

**Keywords:** Endometrial Cancer, Germline variants, Whole exome sequencing, DiscovEHR, TCGA, Uterine Cancer

## Abstract

**Background:**

Endometrial cancer (EMCA) is the fifth most common cancer among women in the world. Identification of potentially pathogenic germline variants from individuals with EMCA will help characterize genetic features that underlie the disease and potentially predispose individuals to its pathogenesis.

**Methods:**

The Geisinger Health System’s (GHS) DiscovEHR cohort includes exome sequencing on over 50,000 consenting patients, 297 of whom have evidence of an EMCA diagnosis in their electronic health record. Here, rare variants were annotated as potentially pathogenic.

**Results:**

Eight genes were identified as having increased burden in the EMCA cohort relative to the non-cancer control cohort. None of the eight genes had an increased burden in the other hormone related cancer cohort from GHS, suggesting they can help characterize the underlying genetic variation that gives rise to EMCA. Comparing GHS to the cancer genome atlas (TCGA) EMCA germline data illustrated 34 genes with potentially pathogenic variation and eight unique potentially pathogenic variants that were present in both studies. Thus, similar germline variation among genes can be observed in unique EMCA cohorts and could help prioritize genes to investigate for future work.

**Conclusion:**

In summary, this systematic characterization of potentially pathogenic germline variants describes the genetic underpinnings of EMCA through the use of data from a single hospital system.

**Electronic supplementary material:**

The online version of this article (10.1186/s12920-019-0504-9) contains supplementary material, which is available to authorized users.

## Background

Endometrial cancer (EMCA) is the most common cancer of the female reproductive tract with an estimated 62,230 new cases and 11,350 deaths estimated in 2018 in the United States alone [1]. The treatment of EMCA has become a major issue for the health-care system because of its increasing incidence and death rate over the past two decades [2]. Traditional U.S. categorization of EMCA is based into two broad classifications from the National Comprehensive Cancer Network (NCCN), type 1 and 2, based on histology, steroid hormone receptor expression, and prognosis [3]. Type 1 is more common, and is characterized by endometrioid histology, is estrogen potentiated, estrogen receptor (ER) and progesterone receptor (PR) positive, and generally carries a favorable prognosis [2–4]. Type 2 is ER/PR negative and carries a much poorer prognosis [4]. Epidemiological studies confirm an association of EMCA with obesity [5], early menarche, late menopause, nulliparity, exogenous factors (estrogen only use), and other lifestyle factors related to low physical activity [3, 6]. While type 1 is treated by surgery followed by radiation for high risk features, type 2 is treated by radiotherapy or surgery followed by systemic chemotherapy. Nevertheless, treatment has high variability in efficacy and side effects [7]. Of those diagnosed with endometrial cancer, 90% is sporadic while the remaining 10% is hereditary [8]. Lynch syndrome or hereditary nonpolyposis colorectal cancer (HNPCC), is an autosomal dominant disorder that not only represents an increased risk of colon cancer, but an increased risk also of EMCA for women [8]. Additionally, it is characterized by a mutation in one of a group of DNA mismatch repair genes (*MSH1*, *MSH2*, *MLH6*, *PMS2* or *EpCAM*) [9, 10]. However, not all families that meet clinical criteria for Lynch syndrome have an identifiable mutation in these genes [11].

DNA sequencing can identify genetic variants associated with different types of cancer. For most cancers, EMCA included, somatic mutations and matched controls have been used to generate insights as to the potential pathogenicity of the variants (Single Nucleotide Variants (SNVs) and insertions/deletions (indels)) [12–15]. A motivating factor behind studying the germline is supported by the “two hit hypothesis” [16], which describes when a tumor suppressor gene is inactivated initially by a germline mutation followed by a somatic mutation on another allele that leads to tumorigenesis. While there remain open questions related to this hypothesis, the investigation of loss of heterozygosity (sometimes referred to as “allelic loss”) of a tumor suppressors has provided support for this theory [17–20]. Furthermore, the analysis of germline variants has improved the detection of driver mutations when somatic variants have also been available [21, 22]. The Cancer Genome Atlas (TCGA) illustrated that cancer susceptibility genes could be identified across several cancer types using data produced from the germline, including but not limited to EMCA, by searching for enrichment of rare variants that resulted in truncations [23]. Moreover, germline data has been used to identify genes previously unknown to be associated with ovarian cancer [24]. Thus, investigating germline variants serves as a tool to assist in the characterization of potential genetic drivers underlying cancer.

Though a number of studies have generated significant findings related to the underlying genetic architecture of EMCA using only germline or matched samples [22, 25, 26], there are growing number of hospital-systems and country-wide genetic studies that have primarily generated germline level data [25]. These projects offer new ways to potentially investigate EMCA. For instance, our group and others have reported the use of  patient-participant billing codes extracted from electronic health records (EHR) and common variants from patient-participants to perform association studies [26–28]. A recent study identified predisposition mutations in an EMCA cohort using a multiplex PCR panel [29]. However, it is largely unknown if comparing rare germline variants from whole exome sequencing between case and control cohorts from a single institution can reproduce what is known and identify novel genetic underpinnings related to EMCA, however it is unmistakable that this strategy could be used by a variety of institutions and in other disease contexts.

In 2007, Geisinger Health System (GHS) launched MyCode, a system-wide biobanking program to link samples and electronic health record (EHR) data for broad research use [30]. GHS is an integrated health system, serving a stable patient population, and with longitudinal EHR data that documents patients’ treatment and clinical outcome [30]. These features of MyCode can be used to compare genetic variants in individuals with and without cancer in a large clinical population. Recently, we reported the results of analysis of more than 50,000 MyCode DNA samples that had undergone whole exome sequencing (WES) as part of the DiscovEHR study [31]. Among DiscovEHR participants were 297 patient-participants who had been diagnosed with EMCA. We hypothesized that characterization of germline variants in WES data in a cohort of participants with EMCA would lead to insights into the genetic basis of EMCA. In this study, we describe the identification of rare, potentially pathogenic variants in DiscovEHR EMCA, a non-cancer cohort, and other hormone related malignancy cancer cohort from a single hospital system.

## Methods

### Patient-participant cohorts

This study consisted of GHS patients who consented to participate in the MyCode Community Health Initiative [30]. MyCode participants agree to provide samples for broad research use and linking of samples to data in the EHR database as part of the DiscovEHR study. EMCA participants were identified through ICD-9 code and then validated through manual chart review of the pathology report (*N* = 297). Demographic information including age and BMI at the time of diagnosis, histology, stage, treatment and overall survival were obtained from the EHR by a Gynecologic oncologist. The elderly non-cancer control cohort (NCC, *N* = 2120) consisted of females older than 70 years old, with no history of cancer diagnosis (absence of ICD9/ICD10 encounter/problem list diagnosis codes related to cancer). The other hormone related malignancy (OHRM) cohort (*N* = 1463) was generated by identifying female participants in DiscovEHR who have ICD-O (international classification of diseases for oncology) codes related to breast (c50x) or ovarian cancer (C48.0, C48.1, C48.2, and C56.9). For further analysis, stages 1 and 2 were defined as “early”, while 3 and 4 EMCA were defined as “late”. The FIGO and TNM staging systems were used. U.S. treatment guidelines were from the National Comprehensive Cancer Network (NCCN) [3].

### Exome sequencing and variant calling

WES data was collected from MyCode participants from the DiscovEHR database and processed with slight modifications [31]. Raw reads were aligned using BWA-mem. Mapping and alignment to GRCh37.p13 was then performed and variants were called using Genome Analysis Toolkit (GATK) “best practices” [32]. Described in brief, after indel realignment, and base recalibration using BQSR (Base Quality Score Recalibration) for the entire GHS cohort, and gVCFs (genomic VCF) are called. The gVCF files were then combined into a merged gVCF and the recalibrated haplotypes are then called using GATK HaplotypeCaller. These gVCFs were then filtered for high quality variants using variant quality score recalibration (VQSR). Another filtering step was used to obtain variants with genotype quality ≥ 20 [33].

### Variant filtration and annotation

Participant VCFs were selected from the DiscovEHR study project that were associated with each cohort. Additional file 1: Figure S1 summarizes the process by which variants were characterized as potentially pathogenic. Variants that fell within the genomic boundaries (RefSeq annotations using VEPv91) of genes (635 unique genes) from the TARGET database (https://software.broadinstitute.org/cancer/cga/target (version 3)) and/or Cancer Gene Census (CGC, https://cancer.sanger.ac.uk/census (downloaded on March 7th, 2017)) were carried forward [34]. Variants were then filtered based on their likelihood of being pathogenic [35–37]. Those variants with 1 or more star or called as either “likely pathogenic” or “pathogenic” in Clinvar [35] or were “High” in VEP (Variant Effect Predictor) were also included [36]. Variants with a minor allele frequency greater than 1% in either the DiscovEHR cohort, Exome Aggregation Consortium (EXaC [38]), NHLBI GO Exome Sequencing Project (ESP [39]) and 1000 Genomes Project were removed [40]. This procedure was based on recommendations from the American College of Medical Genetics and Genomics [41]. Variants that made it through this pipeline were then included in downstream analysis.

To reformat, summarize, and visualize the data, the following R packages were used: ggplot2, dplyr, tidyr, GenVisR, and reshape2. Typically, only one transcript isoform made it through the variant filtration and annotation pipeline. Variants were grouped into three categories, synonymous, non-synonymous, and predicted loss of function (pLoF) for further analyses. These definitions adhere closely to previous work [31]. If the VEP consequence was synonymous_variant the variant was categorized as “synonymous”. If the VEP consequence was missense_variant, stop_retained_variant, initiator_codon_variant, inframe_deletion, inframe_insertion, or splice_region_variant the variant was categorized as “non-synonymous”. If the VEP consequence was stop_gained, stop_lost, start_lost, splice_donor_variant, splice_acceptor_variant, frameshift_variant, disruptive_inframe_deletion, disruptive_inframe_insertion, or protein_protein_contact the variant was categorized as “pLoF”. If the splice_region_variant co-occurred with a synonymous, missense, or pLoF variant it was called as synonymous, missense, or pLoF, respectively. If the variant was located in lower confidence region (e.g. UTR, upstream_gene_variant, downstream_gene_variant, intron_variant, or TF) the variant was excluded from analysis. Variants were excluded if they were not targeted by probes in the exome-capture process. The average EMCA patients with a variant per control group patient was calculated for each gene by: (Variants_GeneX in EMCA patients_/297 EMCA patients)/ (Variants_GeneX in control patients_/2120 control patients). The average OHRM patients with a variant per control group patient was calculated for each gene by: (Variants_GeneX in OHRM patients_/1486 OHRM patients)/ (Variants_GeneX in control patients_/2120 control patients). Graphical and statistical analysis was performed in R.

### Comparison to TCGA Germline Uterine Cancer data

Uterine Corpus Endometrial Carcinoma (UCEC) germline and somatic variants were retrieved from the Broad GDAC Firehose (https://gdac.broadinstitute.org). The germline VCFs were processed in the same manner as the DiscovEHR data (see previous section) to identify variants in the list of genes from TARGET and CGC (Additional file 1: Figure S1).

## Results

### Demographics and histology of cancer and non-cancer cohorts

The Geisinger MyCode community health initiative includes over 150,000 participants who have agreed to provide blood samples for broad research use [30] (www.geisinger.edu). In the first 50,726 participants to undergo WES analysis, we identified 297 patients with a diagnosis of EMCA (Additional file 5: Table S1). The average age of individuals with EMCA in our study was 61, which is similar to the national average, e.g. 60 years old (cancer.org). While most women diagnosed with EMCA in the U.S. are older than 45, the age range is from 27 to 87 in this study. However, 94% of our samples came from individuals older than 45, suggesting our cohort represents a similar age as is seen nationally. The DiscovEHR cohort as a whole has an average BMI of 30 kg/m^2^ [31], and the average BMI in the EMCA cohort was 38 kg/m^2^ (Additional file 5: Table S1). This result is consistent with previous research which has found an increased risk of uterine-related cancer with increasing BMI [42].

Chart review revealed that most EMCA cases were of endometrioid histology, and early stage and grade (Additional file 5: Tables S2-S5). An elderly female non-cancer cohort (NCC, *N* = 2120) was used to identify potential EMCA-associated genetic variants by selecting older individuals who have no record of cancer. Additionally, a separate hormone related malignancy (OHRM) cancer cohort (*N* = 1463), consisting of individuals with breast or ovarian cancer, was also analyzed. Since the OHRM cohort represents estrogen-driven cancer etiology similar to EMCA, it provides an opportunity to identify EMCA-specific variants as opposed to other hormone-related cancers. Our control cohort has an average BMI of 27 kg/m^2^ which while overweight, is on average lower than the entire DiscovEHR cohort as a whole. The average BMI of the OHRM cohort is 29 kg/m^2^, which is close to an obese classification, and consistent with the association between obesity and cancer [43]. The total number of patients with endometrioid EMCA was 265, while 2 had non-endometrioid EMCA that are grade 3 (Additional file 5: Tables S2 and S4). The DiscovEHR and TCGA cohorts had similar distributions of grade 2 samples, but TCGA had many more grade 3, and fewer grade 1 (Additional file 5: Table S4). Grade one and two U.S. estimates were similar to the other cohorts [44]. Compared to U.S. estimates, TCGA has more grade 3 as a percentage and DiscovEHR had a smaller percentage (Additional file 5: Table S4). Downstream analysis of all grades and stages are performed together unless otherwise noted. The average time of follow up after diagnosis was 6 years, and most patients were disease free in the absence of further therapy (Additional file 5: Tables S1 and S5). All patients who were stage 3 or 4 received surgery (Additional file 5: Table S5).

### Identifying rare pathogenic germline variants in cancer and non-cancer cohorts

To identify variants that are relevant to EMCA a bioinformatics pipeline was created to identify variants that are predicted to be likely pathogenic or pathogenic (Additional file 1: Figure S1). Only genes that were in the TARGET or Cancer Genome Census (CGC) database (635 genes total) were included to increase confidence that variants we identify are related to cancer or therapeutic outcomes [34, 45]. Variants were binned into the 635 TARGET and/or CGC genes, then annotated using ClinVar and variant effect predictor (VEP) to identify likely pathogenic or pathogenic variants. Only variants with minor allele frequency (MAF) less than 1% in the Geisinger DiscovEHR population, and not greater than 1% in any total or sub population from Exome Aggregation Consortium (ExAC [38]), Exome Sequencing Project (ESP [39]), or 1000 genomes project [40] were included. This process identified variants in 28 to 32% of the participants across the three cohorts (Table 1). The number of total and unique variants across the three cohorts is summarized in Table 1. Therefore, all variants described from here forward are considered likely pathogenic or pathogenic.

**Table 1:** Summary of rare pathogenic variant distribution across cohorts

	EMCA	NCC	OHRM
Participants^a^	297	2120	1486
Participants with variants after filter^b^	85 (28.6%)	628 (29.6%)	462 (31.1%)
Genes with variants after filter^c^	62	211	205
Loci with variants^d^	73	485	371
Rare variant burden across participants in each cohort^e^	99	791	593

**Fig. 1:**
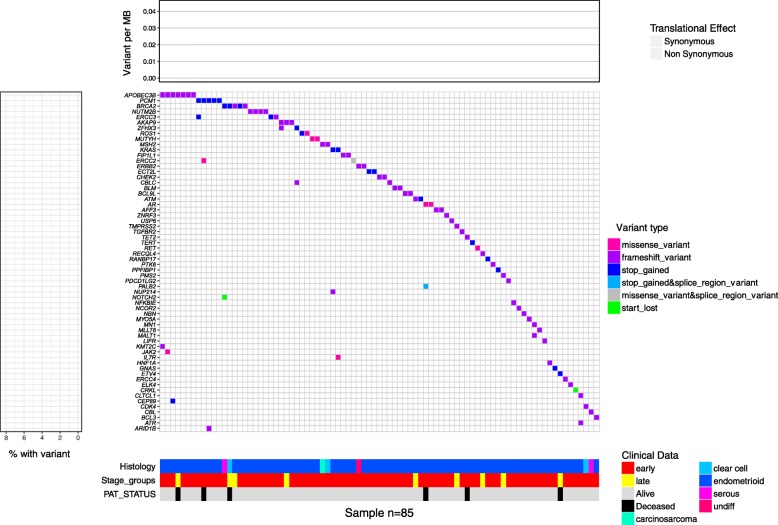
Waterfall plot of all genes with pathogenic variants. Waterfall plot of all EMCA samples that contained rare variants that passed the filter from Additional file 1: Figure S1. The main heatmap contains columns which represent an individual participant (*N* = 86), and rows that represent genes, while the color that fills in the cell represents the type of variant present for a specific participant in a specific gene. The heatmap below illustrates that histology, cancer stage and patient survival status, each column represents a different participant. “Undiff” refers to undifferentiated histology. The graph to the left shows the percentage of participants who have a rare variant in a gene, relative to all participants with variants, while the bar plot above the main graph represents the variant burden for each participant

Summary level data of participants from WES and rare variant analysis. The total number of participants included in each cohort (^a^). The total number of participants from “a” that had at least one rare variant that met criteria from Fig. 1 workflow, (^b^). The number of genes with at least one rare variant that met workflow criteria (^c^). The number of unique variants present after filtering using the bioinformatics pipeline in Fig. 1 (^d^). The total number of unique and non-unique rare variants present across the participants in the cohort (^e^)

### Exploring EMCA histology and rare variants

The variants were visualized in a participant centric graph using a co-mutation plot, sometimes referred to as a waterfall plot (Fig. 1). There did not appear to be enrichment of a specific EMCA histology, and/or patient status among genes that are often mutated (i.e. *APOBEC3B*) compared to those with few variants (i.e. *ARID1B*) (Fig. 1). Generally, most participants had variants associated with the most common diagnosis (i.e. endometrioid histology). Of the 297 participants with EMCA, six had been previously clinically diagnosed with Lynch syndrome, however, only two contained a rare pathogenic variant that met our workflow criteria (Additional file 2: Figure S2). The participants had a frameshift variant in *MSH2*, a Lynch syndrome gene [46].

### Characterization of pathogenic germline variants between EMCA, OHRM and NCC cohorts

**Fig. 2:**
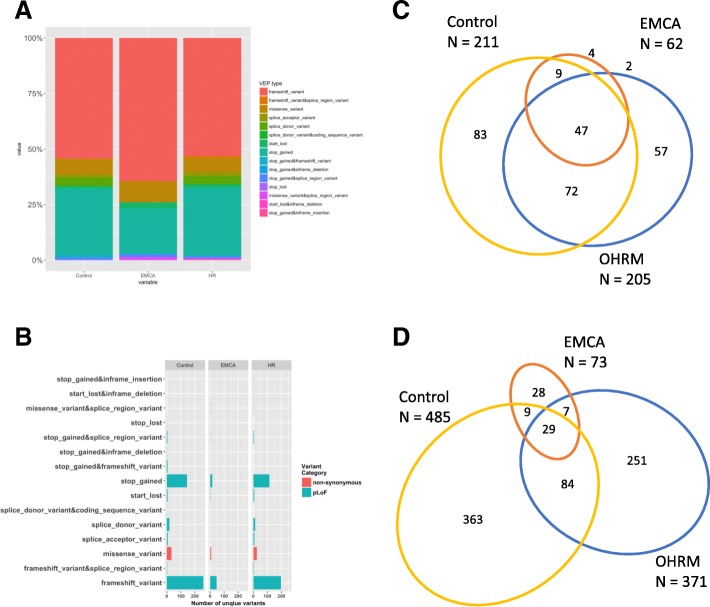
Distribution of types of rare variants across cohorts. The total number of unique variants are represented by their VEP annotation. Additionally, each type of variant is grouped by the variant category (**a**). The percentage of each variant category represented in each cohort (**b**). The overlap between genes with variants represented in each cohort (**c**). The number of unique loci (variants) that overlap between each cohort (**d**)

The variants across the three cohorts included a mixture of indels and single nucleotide variants (SNVs) (Fig. 2a). Based on criteria (see methods) from previous work, SNVs and indels were grouped together based on whether they were synonymous, non-synonymous or variants that are predicted to cause a loss of function (pLoF). No synonymous variants were identified as potentially pathogenic. Most of the non-synonymous variants were missense, while most pLoF variants were stop gained or frameshift (Fig. 2b). Non-synonymous and pLoF made up similar percentages of the variants in all three cohorts (Fig. 2b). In a previous analysis of variants from more than 50,000 DiscovEHR participants, non-synonymous and synonymous variants made up a greater number of sites with variants than did pLoF [31]. However, after our rare variant annotation algorithm (Additional file 1: Figure S1) was applied to the cohorts in this study, synonymous variants were filtered out leaving mostly pLoF and some non-synonymous variants (Fig. 2a). These results demonstrate that the algorithm enriches for rare variants with potential pathogenic influence.

The number of unique variants and genes that contained variants are described in Table 1. The genes overlapping between the 3 cohorts is shown in Fig. 2c. While there was considerable overlap between the cohorts, 4 genes were unique to the EMCA cohort (e.g. *CDK4, LIFR, MALT1,* and *MSH2*). Two genes were identified only in the EMCA and OHRM cohorts (e.g. *PMS2* and *TMPRSS2*). A comparison of unique variant loci showed differences among the cohorts compared to gene-wise comparison (Fig. 2d). The vast majority of genes present in the EMCA cohort could be found in the other two cohorts. This result suggests that to identify more genes with EMCA relevant genetic variation, an alternate metric such as calculating the difference in frequency between the genes with rare pathogenic variants in EMCA and the other cohorts could be useful.

### Comparing rare variant burden between cancer and NCC cohorts

**Fig. 3:**
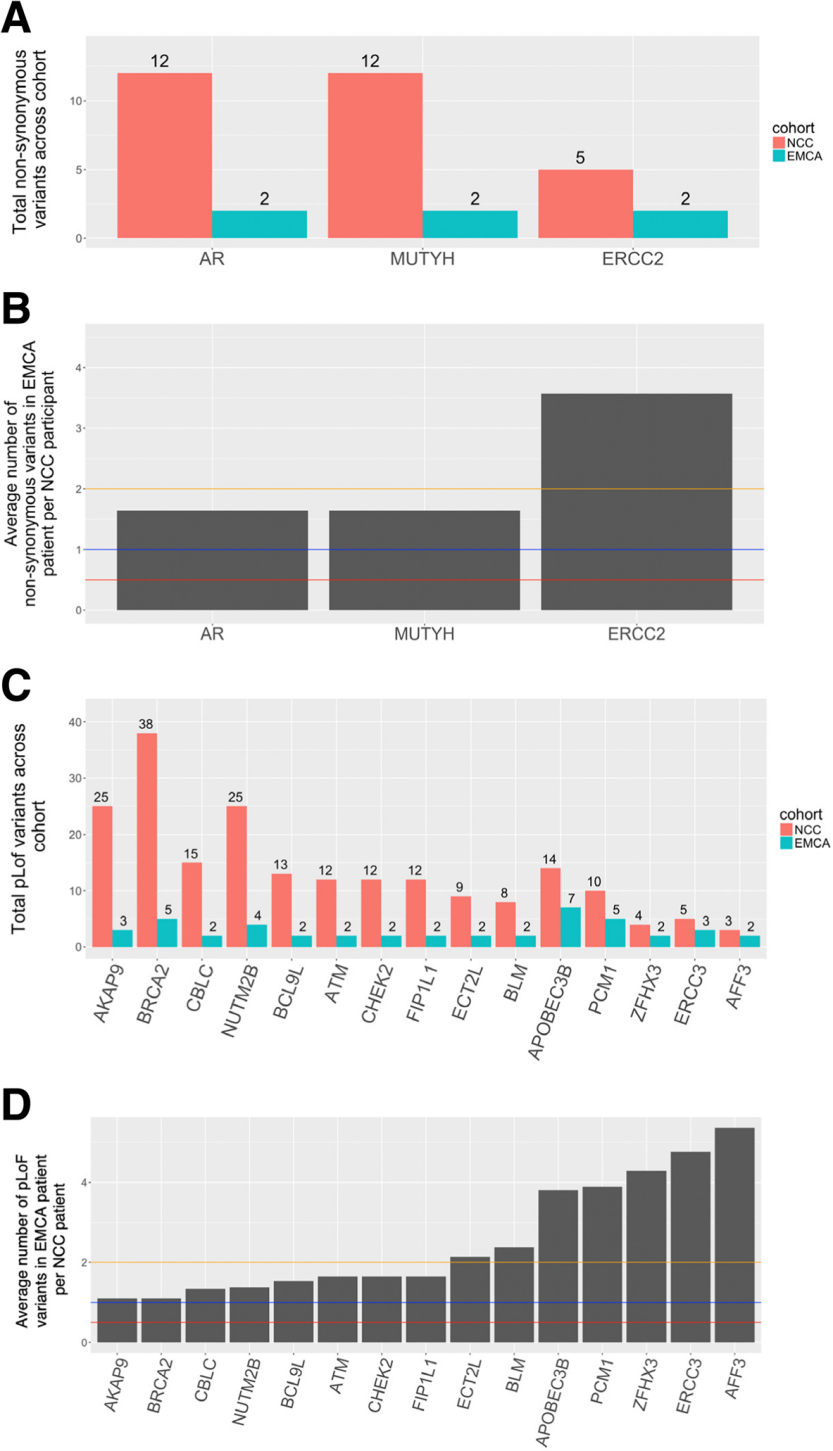
Non-synonymous and pLoF variants among EMCA to non-cancer control cohort. **a** For each gene with at least two variants in both EMCA and NCC, the ratio of non-synonymous variants across the EMCA cohort was divided by those in the NCC after adjusting for differences in cohort size. Orange, blue and red lines are used to delineate 2, 1 and 0.5 fold EMCA burden relative to the NCC cohort. **b** The total number of rare non-synonymous variants from each cohort for each gene. **c** and **d** the same as **a** and **b**, respectively, except pLoF variants were used

We next evaluated whether analysis of variant burden could identify genes that are more likely to be associated with EMCA. The number of variants per individual was compared in the EMCA and NCC cohorts (Table 1) (see methods). Only genes with at least two variants across the EMCA and NCC cohorts were considered. There was only one gene, *ERCC2,* with a higher burden of non-synonymous variants in EMCA participants compared to the NCC cohort (Fig. 3a and b). Seven genes showed at least a 2-fold enrichment in the EMCA cohort compared to NCC using pLoF variants (Fig. 3c and d; *ECT2L*, *BLM*, *APBEC3B*, *PCM1*, *ZFHX3*, *ERCC3*, and *AFF3*). To verify that the increased burden among EMCA participants is specific to EMCA and not hormone related cancers, the burden between OHRM and the NCC cohort was also measured (Additional file 3: Figure S3 and Additional file 4: Figure S4). Only *RNF213* was found to have increased pLoF variant burden in the OHRM cohort, however this was not observed in the EMCA cohort (Additional file 4: Figure S4 and 3D). Since there were no shared genes with at least 2-fold enrichment among EMCA and OHRM relative to the NCC, all of the genes with high variant burden in the EMCA cohort are unique to EMCA compared to the OHRM cohort. We used a difference of proportions test to evaluate the differences between EMCA and NCC or OHRM and NCC (Additional file 6). While several genes had a *p*-value less than 0.05, they were not significant after correcting for multiple tests. These results suggest a larger sample size or different experimental design is necessary for capturing statistically significant results (Additional file 6).

### DiscovEHR germline variants are reproduced in TCGA study

**Fig. 4:**
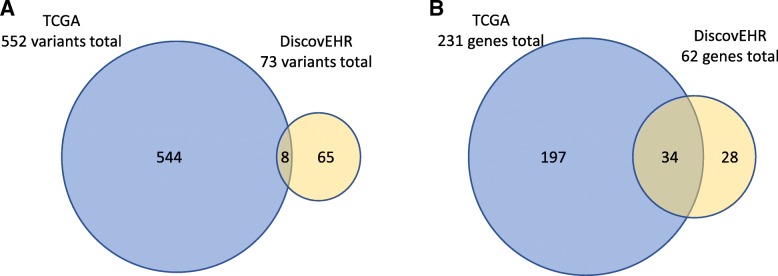
Overlap between DiscovEHR and TCGA germline variants from EMCA and uterine cancer samples. Rare potentially pathogenic variants were identified from the germline uterine cancer cohort in TCGA. The overlap between TCGA variants and those from this study (DiscovEHR) is illustrated in the Venn diagram (**a**). The overlap of genes from TCGA and the genes with variants from this EMCA cohort from this study (**b**)

We also compared germline variants in the DiscovEHR EMCA cohort to the uterine cancer samples in the Cancer Genome Atlas (TCGA) Research Network. TCGA performed a comprehensive, multiplatform analysis of type 1 and 2 EMCA using array- and sequencing-based technologies, including WES [47]. DiscovEHR and TCGA had 89 and 81% endometrioid histology, respectively, however median follow-up time was much greater for the DiscovEHR EMCA cohort (67 months) relative to TCGA (32 months) [47]. A total of 553 rare variants from TCGA germline variants met the criteria of the workflow in Fig. 1 that was applied to the EMCA DiscovEHR data (Fig. 4). Of these likely pathogenic variants, only eight were also found in the DiscovEHR EMCA cohort (Fig. 4a). The 73 Geisinger Health System germline variants were in 62 different genes, 34 of which were also identified in the TCGA EMCA cohort (Fig. 4b). Of the eight genes that had higher burden in the EMCA cohort relative to NCC, four (e.g. *BLM*, *ECT2L*, *ERCC2*, and *ERCC3*) were present in the 34 genes represented in both DiscovEHR and TCGA. Additionally, *MALT1* and *MSH2*, two genes that were only observed to have variants in the EMCA only cohort (Fig. 2c) were among the 34 genes that replicated. These results support the concept that rare variants binned into genes can do a better job of capturing replication relative to being located in the same loci across individuals [48]. Furthermore, the relatively high congruency between the two studies at the gene level highlights how germline variation in EMCA participants is reproducible for this study. Only one variant (e.g. nonsense variant in the gene *ETV4*) was found to replicate between the Geisinger germline results and the TCGA somatic mutations.

## Discussion

While significant progress has been made identifying somatic variants associated with EMCA [47, 49], a number of studies have pointed to the usefulness of looking at germline variants as an alternative means to characterize the genetic etiology of EMCA [29, 50]. In this work, we describe the landscape of germline variants from participants diagnosed with EMCA using the WES data from the DiscovEHR study. We identified rare variants in 4 genes that were unique to the EMCA cohort. Rare variants in the genes *CDK4, LIFR, MALT1,* and *MSH2* were identified in the DiscovEHR EMCA cohort but not NCC and OHRM. *CDK4* promotes progression of the cell cycle and increased expression is observed in 34–77% of endometrioid endometrial carcinoma (EEC) [51]. Additionally, the specific activity of CDK4/6 has been illustrated to be a biomarker for predicting recurrence of EEC in pathologically low-risk group of patients [52]. Recent evidence suggests the expression of leukemia inhibitory factor receptor (*LIFR*) affects multiple signaling pathways in the endometrium of patients with adenomyosis during the window of implantation for in vitro fertilization [53, 54]. Chromosomal translocation of *MALT1* associated with MALT lymphoma is often restricted to the endometrium [55]. Individuals with germline mutations in *MSH2* can be diagnosed with Lynch Syndrome, which in turn is associated with a high risk of colorectal cancer, including but not limited to EMCA [56]. Since an extensive literature exists on all but *MALT1*, it suggests further work should be devoted to teasing out its connection to EMCA.

*APOBEC3B* was the gene most often identified as having a rare variant among EMCA participants. *APOBEC3B* is a member of a gene family, which consists of seven members, that cause cytosine-to-uracil deamination of single-stranded DNA [57]. These cytosine deaminases mediate intrinsic immunity to retroviruses and endogenous retrotransposons [58, 59]. Mutation or low expression of the tumor suppressor p53 is associated with an increase in expression and activity of *APOBEC3B* in endometrial cancer and other types of cancer as well [57]. *PCM1 and BRCA2* were found to have the second most rare variants among EMCA participants (*N* = 5 each). *PCM1* codes for a protein that is responsible for anchoring microtubules to centrosomes and has previously been associated with thyroid cancer, leukemia, and T-cell lymphoma [60–63]. However, *PCM1* does not have a previously known connection to EMCA, suggesting it is a novel candidate for investigating its relationship to EMCA. Alternatively, previous work found carriers of *BRCA* mutations, especially *BRCA1*, had increased risk of EMCA [64].

Our data also suggests that relying only on the EMCA cohort or looking for variation that does not overlap with other cohorts to investigate the genetic etiology of EMCA is problematic, as several genes can be missed. Whereas a burden-based approach appeared to be a superior method for finding genes that have EMCA-associated variation. Only four genes were identified as having variants unique to the EMCA cohort, whereas the burden-based approach identified eight genes, and importantly, controlled for genes which have many variants in the NCC or OHRM cohorts. Together, these results suggest a burden-based approach can lead to the identification of more genes that help characterize germline variation and account for genes that are more prone to germline variants in EMCA patient-participants. Several genes that were identified using the burden-based approach regulate DNA repair or transcription. For instance, *ERCC2* and *ERCC3* are a part of the general transcriptional machinery TFIIH and nucleotide excision repair [65]. The transcription factor *AFF3* associated with lymphoid development and neuronal differentiation [66, 67]. *ZFHX3* is also a transcription factor, it acts as a tumor suppressor in multiple cancer types [68, 69]. Though these observations are consistent with a previous body of work which has found a connection between developmental processes and cancer [70], we found that rare variants in these genes may be acutely important for understanding EMCA.

To evaluate how reproducible and biologically relevant the rare variants are among EMCA cohorts we compared the variation observed in our cohort to that were seen in TCGA germline samples. This comparison illustrates consistent variation at the gene level, but not at the variant level. Moreover, these results support a known feature of genetic variation, that while rare variants are infrequently observed at the same exact loci across individuals, trends do appear once the rare variants are analyzed in the context of a gene or pathway [48]. Replication is especially important to look for here since rare variants may be spurious [48]. Thus, our ability to find reproducible variation increases confidence that variation within genic regions will be helpful for characterizing EMCA on a molecular level. The genes identified may play a role in the development of EMCA, therefore this work may provide a useful strategy for identifying possible therapeutic targets either through drug or mutation. While the EMCA cohort from DiscovEHR had a similar distribution of stages, there were more grade 3 samples in TCGA. Thus, when comparing the two, the interpretation should consider that the overlap was between samples of varying stages and grades. As discussed below, larger sample sizes in the future may allow for stratified analyses. Nonetheless, because most EMCA diagnoses in the U.S. are estimated to be grade 1/2 and stage 1, it is possible that results from DiscovEHR can be generalized.

Previous work has demonstrated how individual germline variants contribute to EMCA [49]. Here we utilized WES data obtained from participants of a single health system as part of the DiscovEHR study. By performing a manually curated chart review, there was added confidence in the identification of those diagnosed with EMCA. An advantage of performing the study using the DiscovEHR cohort is that due to the location, the participants have similar demographics, suggesting that ancestry and socio-economic status likely play a smaller role in explaining variation between participants in the DiscovEHR cohort. Conversely, future studies with larger cohorts that have less homogenous ancestry and cancer types could provide important insights into the etiology of EMCA across different subpopulations. A larger cohort, with more individuals across ancestries, stages, treatments, and outcomes could also provide a useful platform for characterizing our genetic understanding of EMCA and performing more risk assessment related analyses.

An outstanding obstacle in the field of EMCA research is the difficulty in risk assessment for individuals who already have EMCA. While this work did not address that issue, we look forward to future work that applies the use of EHR and WES to that specific clinical application. For instance, obesity is a risk factor, but it could also independently be associated with the variant burden. Future studies will need to be carefully designed in order to test for this effect. Having said that, the genes identified in this study may play a role in the initiation or inception of EMCA, and with experimental functional validation could be prioritized as potential therapeutic targets. In summary, this study suggests that WES from a single hospital system can provide useful insights into the molecular signatures for which to distinguish variation in EMCA from that in NCC and OHRM cohorts. Moreover, these genes and variants may help identify causal links to the pathogenesis of EMCA.

## Conclusions

The purpose of this study was to investigate the differences and similarities between potentially pathogenic germline variants among patient participants from EMCA, OHRM, and NCC cohorts using EHR. Although larger sample sizes or alternative approaches will be needed to capture statistically significant associations, a number of conclusions can be made from this analysis which characterize EMCA in new ways. Binning potentially pathogenic variants from the DiscovEHR WES data into genes illustrated greater overlap between cohorts compared to looking at the overlap of loci. Only four genes had variants unique to the EMCA cohort, where as a burden-based approach detected eight genes that were enriched with potentially pathogenic variants. High concordance between the DiscovEHR and TCGA cohorts illustrates that reproducible potentially pathogenic germline variation can be observed in multiple studies. In summary, there are many overlapping genetic features between EMCA and non-EMCA cohorts, however, a burden-based approach can best help to characterize the genetic underpinnings of EMCA.

### Web resources

Variant sites and frequencies with basic annotations from the DiscovEHR study is hosted in the following database and webserver: www.discovehrshare.com. Further information concerning the reproduction of results described in this article is available upon reasonable request and subject to a data use agreement. The TARGET database (https://software.broadinstitute.org/cancer/cga/target (version 3)) and Cancer Gene Census (CGC, https://cancer.sanger.ac.uk/census (downloaded on March 7th, 2017)) were used in this work. Uterine Corpus Endometrial Carcinoma (UCEC) germline and somatic variants were retrieved from the Broad GDAC Firehose (https://gdac.broadinstitute.org).

## Additional files

Additional file 1: **Figure S1.** Bioinformatics workflow for detecting pathogenic variants. Variants are first binned into the 635 genes from TARGET and CGC. They are then carried forward if they meet certain criteria from multiple variant annotation databases (e.g. Clinvar and VEP). Finally, variants with a MAF > 1% In the MyCode cohort, or any total or subpopulation from EXaC, ESP and 1000 genomes. (PPTX 43 kb)

Additional file 2: **Figure S2.** Variants in Lynch Syndrome Participants. The main figure is a heatmap of columns for each of the 6 participants who have been previously diagnosed with Lynch syndrome. The rows represent the genes in which these variants reside in and the color is the type of variant (see key to right). The variant burden for each participant and the individual genes are represented has histograms above and to the left of the main figure, respectively. Below the main heatmap is another diagram which illustrates the histology, stage and patient status along with reporting that all 6 participants had a Lynch diagnosis. (PPTX 50 kb)

Additional file 3: **Figure S3.** Non-synonymous variants among OHRM and NCC cohorts. (A) For each gene with two variants in both cohorts, the ratio of non-synonymous variants across the EMCA cohort was divided by those in the NCC after adjusting for differences in cohort size. Orange, blue and red lines are used to delineate 2, 1 and 0.5 fold EMCA burden relative to the NCC cohort. The graph inset represents the raw number of variants at each gene between the OHRM and NCC cohort. (B) The number of rare non-synonymous variants from each cohort. (PPTX 73877 kb)

Additional file 4: **Figure S4.** pLoF variants among OHRM and NCC cohorts. For each gene with two variants in both cohorts, the ratio of non-synonymous variants across the OHRM cohort was divided by those in the NCC after adjusting for differences in cohort size. Orange, blue and red lines are used to delineate 2, 1 and 0.5 fold EMCA burden relative to the NCC cohort. The graph inset represents the raw number of variants at each gene between the OHRM and NCC cohort. (B) The number of rare pLoF variants from each cohort. (PPTX 73879 kb)

Additional file 5: **Table S1.** DiscovEHR and TCGA participant demographic information. **Table S2.** DiscovEHR and TCGA EMCA participant demographic information. **Table S3.** Distribution of stages between races and studies as a percentage*. **Table S4.** Distribution of grades across studies for EMCA*. **Table S5.** Total patient information for therapy and outcomes among DiscovEHR EMCA cohort. (DOCX 18 kb)

Additional file 6: Difference of proportions test. After binning variants into genes, a difference of proportions test was performed across all genes between variants in the EMCA and the control or OHRM cohort. *P*-values were adjusted for multiple tests using FDR. (XLSX 16 kb)

## Abbreviations

EMCA: Endometrial cancer
NCC: Elderly non-cancer control cohort
OHRM: Other hormone related malignancy
TCGA: The cancer genome atlas
WES: Whole exome sequencing
