# Correction to: Systematic characterization of germline variants from the DiscovEHR study endometrial carcinoma population

**DOI:** 10.1186/s12920-019-0523-6

**Published:** 2019-05-22

**Authors:** Jason E. Miller, Raghu P. Metpally, Thomas N. Person, Sarathbabu Krishnamurthy, Venkata Ramesh Dasari, Manu Shivakumar, Daniel R. Lavage, Adam M. Cook, David J. Carey, Marylyn D. Ritchie, Dokyoon Kim, Radhika Gogoi, on behalf of the DiscovEHR collaboration

**Affiliations:** 1 Department of Genetics, Institute for Biomedical Informatics, Perelman School of Medicine, University of Pennsylvania, Philadelphia, PA 19104 USA; 2 Biomedical & Translational Informatics Institute, Geisinger Health System, Danville, PA 17822 USA; 3 Weis Center for Research, Geisinger Medical Center, Danville, PA 17822 USA; 4 Huck Institute of the Life Sciences, Pennsylvania State University, University Park, Pennsylvania, PA 16802 USA; 5 Department of Biostatistics, Epidemiology and Informatics, Perelman School of Medicine, University of Pennsylvania, Philadelphia, USA; 6 Institute for Biomedical Informatics, University of Pennsylvania, Philadelphia, USA

**Miller et al. BMC Medical Genomics (2019) 12:59.**

**https://doi.org/10.1186/s12920-019-0504-9**

Following publication of the original article [1], the authors reported that Fig. 1 was not correctly processed during the production process. The correct Fig. 1 is given below.

The publishers apologise for the inconvenience caused. The original article [1] has been corrected.

**Fig. 1:**
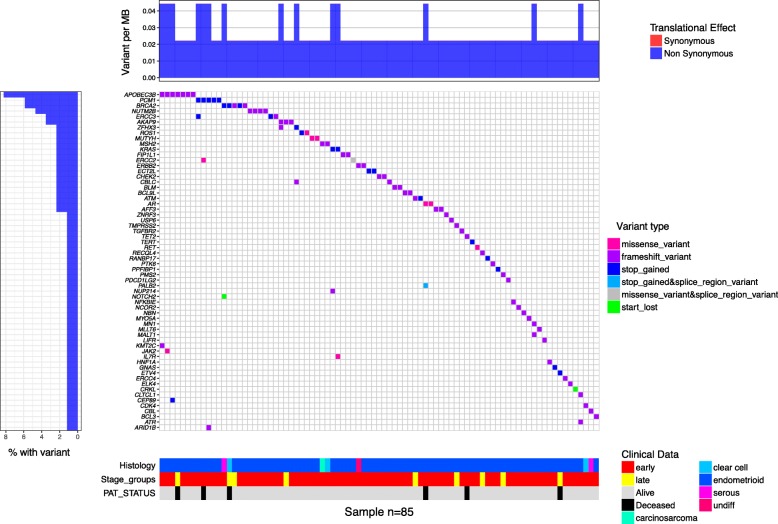
Waterfall plot of all genes with pathogenic variants. Waterfall plot of all EMCA samples that contained rare variants that passed the filter from Additional file 1: Figure S1. The main heatmap contains columns which represent an individual participant (*N* = 86), and rows that represent genes, while the color that fills in the cell represents the type of variant present for a specific participant in a specific gene. The heatmap below illustrates that histology, cancer stage and patient survival status, each column represents a different participant. “Undiff” refers to undifferentiated histology. The graph to the left shows the percentage of participants who have a rare variant in a gene, relative to all participants with variants, while the bar plot above the main graph represents the variant burden for each participant
