# Supplementary material for: Systematic characterization of germline variants from the DiscovEHR study endometrial carcinoma population
Source: BMC Med Genomics. 2019 May 3;12:59. doi: 10.1186/s12920-019-0504-9 (PMC6499978; doi:10.1186/s12920-019-0504-9)
Supplement: Supplementary file 1 — Figure S1. Bioinformatics workflow for detecting pathogenic variants. Variants are first binned into the 635 genes from TARGET and CGC. They are then carried forward if they meet certain criteria from multiple variant annotation databases (e.g. Clinvar and VEP). Finally, variants with a MAF > 1% In the MyCode cohort, or any total or subpopulation from EXaC, ESP and 1000 genomes. (PPTX 43 kb) [file 12920_2019_504_MOESM1_ESM.pptx]

## Slide 1
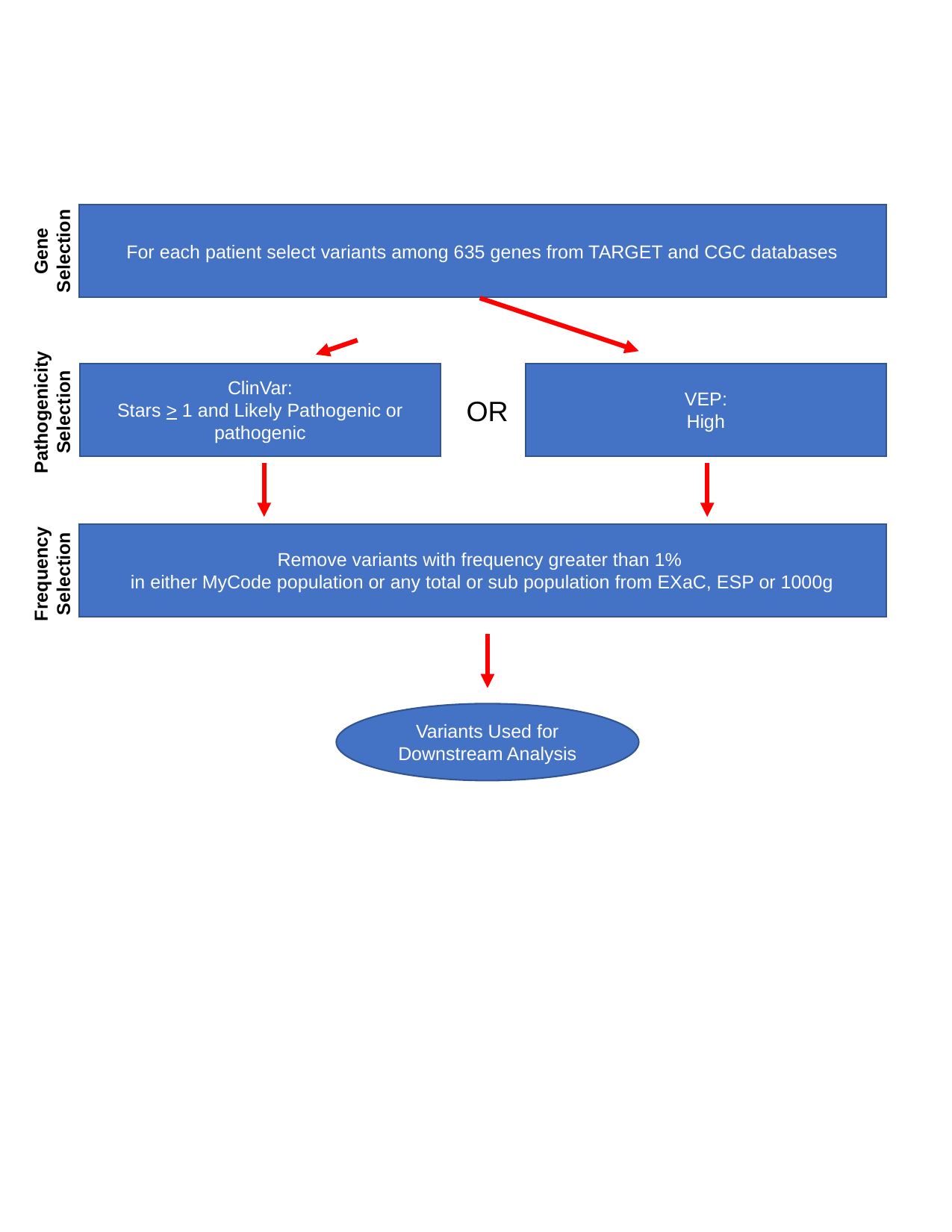

For each patient select variants among 635 genes from TARGET and CGC databases
Gene
Selection
ClinVar:
Stars > 1 and Likely Pathogenic or pathogenic
VEP:
High
Pathogenicity Selection
OR
Remove variants with frequency greater than 1%
in either MyCode population or any total or sub population from EXaC, ESP or 1000g
Frequency
Selection
Variants Used for Downstream Analysis
