# Supplementary figures and images for: Systematic characterization of germline variants from the DiscovEHR study endometrial carcinoma population
Source: BMC Med Genomics. 2019 May 3;12:59. doi: 10.1186/s12920-019-0504-9 (PMC6499978; doi:10.1186/s12920-019-0504-9)

## Slide 1
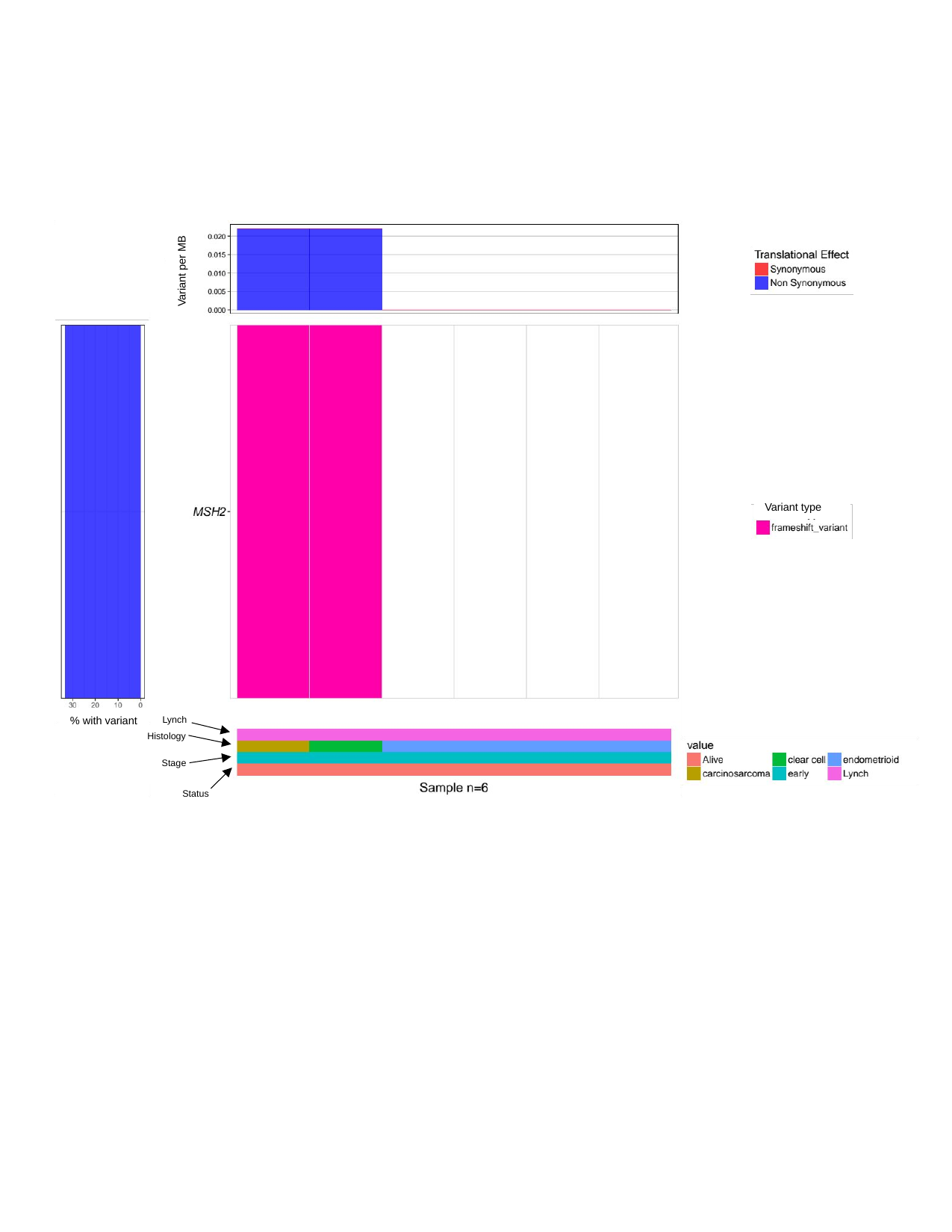

Variant per MB
Variant type
% with variant
Lynch
Histology
Stage
Status

Supplement: Supplementary file 2 — Figure S2. Variants in Lynch Syndrome Participants. The main figure is a heatmap of columns for each of the 6 participants who have been previously diagnosed with Lynch syndrome. The rows represent the genes in which these variants reside in and the color is the type of variant (see key to right). The variant burden for each participant and the individual genes are represented has histograms above and to the left of the main figure, respectively. Below the main heatmap is another diagram which illustrates the histology, stage and patient status along with reporting that all 6 participants had a Lynch diagnosis. (PPTX 50 kb) [file 12920_2019_504_MOESM2_ESM.pptx]

## Slide 1
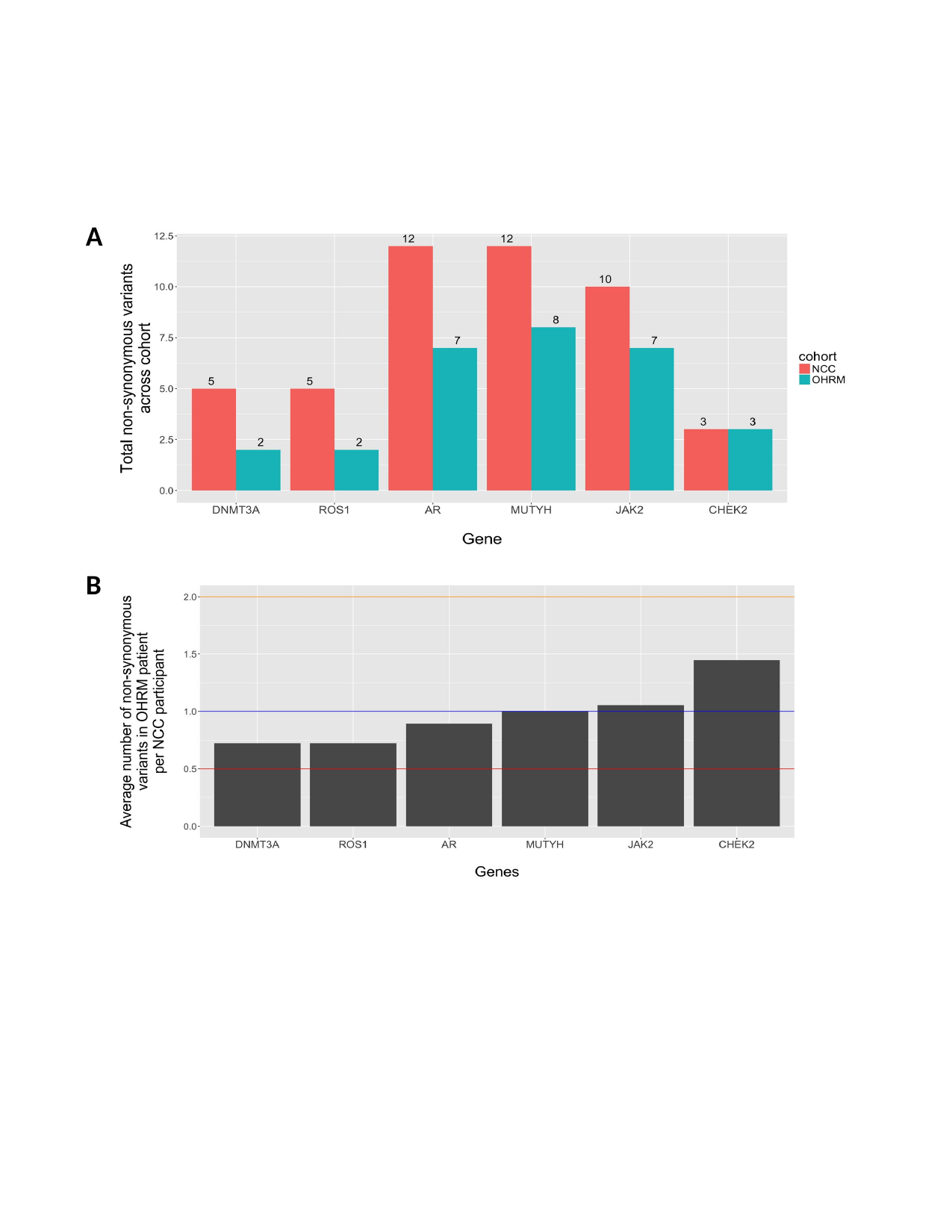

A
B

Supplement: Supplementary file 3 — Figure S3. Non-synonymous variants among OHRM and NCC cohorts. (A) For each gene with two variants in both cohorts, the ratio of non-synonymous variants across the EMCA cohort was divided by those in the NCC after adjusting for differences in cohort size. Orange, blue and red lines are used to delineate 2, 1 and 0.5 fold EMCA burden relative to the NCC cohort. The graph inset represents the raw number of variants at each gene between the OHRM and NCC cohort. (B) The number of rare non-synonymous variants from each cohort. (PPTX 73877 kb) [file 12920_2019_504_MOESM3_ESM.pptx]

## Slide 1
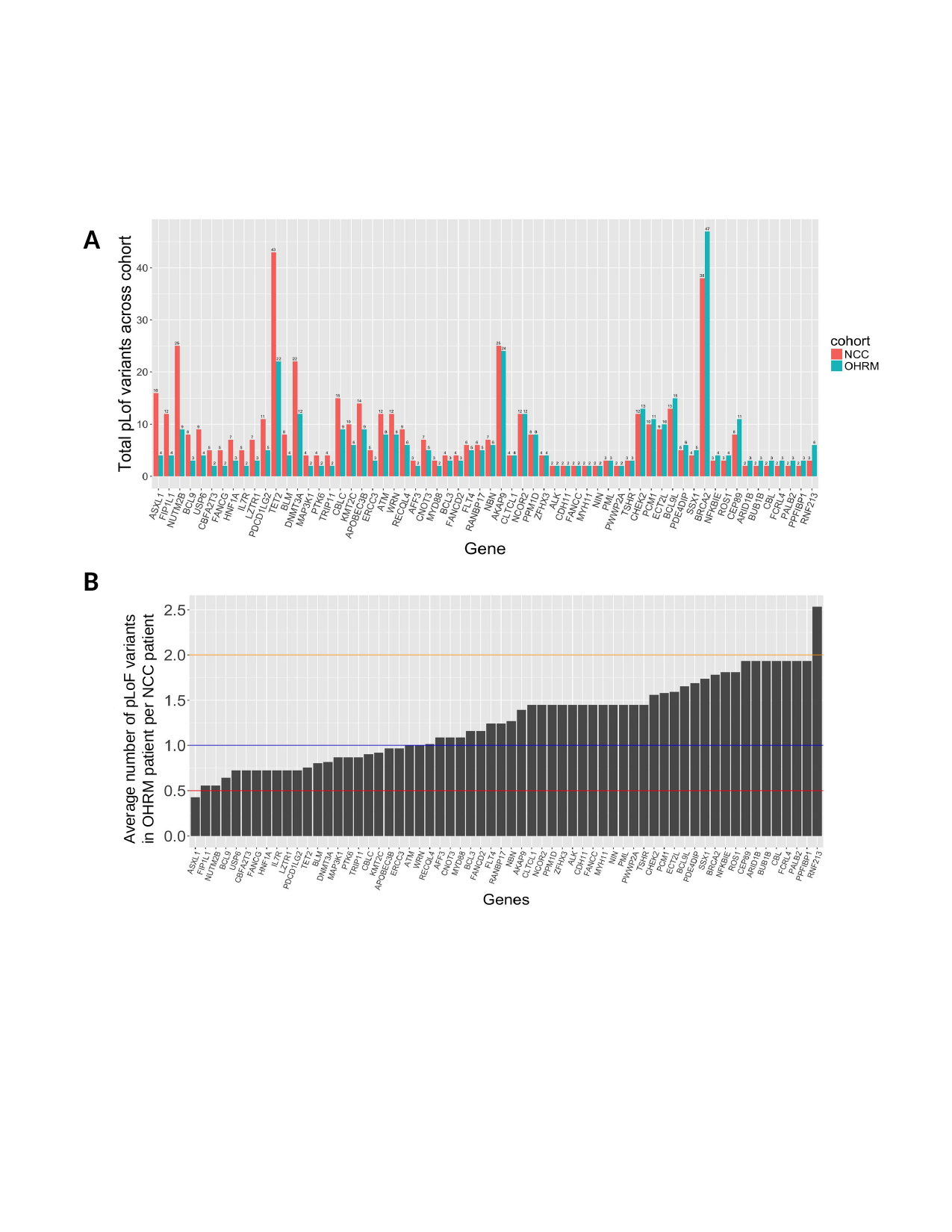

A
B
Average number of pLoF variants
in OHRM patient per NCC patient

Supplement: Supplementary file 4 — Figure S4. pLoF variants among OHRM and NCC cohorts. For each gene with two variants in both cohorts, the ratio of non-synonymous variants across the OHRM cohort was divided by those in the NCC after adjusting for differences in cohort size. Orange, blue and red lines are used to delineate 2, 1 and 0.5 fold EMCA burden relative to the NCC cohort. The graph inset represents the raw number of variants at each gene between the OHRM and NCC cohort. (B) The number of rare pLoF variants from each cohort. (PPTX 73879 kb) [file 12920_2019_504_MOESM4_ESM.pptx]
