# Supplementary material for: Systematic characterization of germline variants from the DiscovEHR study endometrial carcinoma population
Source: BMC Med Genomics. 2019 May 3;12:59. doi: 10.1186/s12920-019-0504-9 (PMC6499978; doi:10.1186/s12920-019-0504-9)
Supplement: Supplementary file 5 — Table S1. DiscovEHR and TCGA participant demographic information. Table S2. DiscovEHR and TCGA EMCA participant demographic information. Table S3. Distribution of stages between races and studies as a percentage*. Table S4. Distribution of grades across studies for EMCA*. Table S5. Total patient information for therapy and outcomes among DiscovEHR EMCA cohort. (DOCX 18 kb) [file 12920_2019_504_MOESM5_ESM.docx]

| **Table S1: DiscovEHR and TCGA participant demographic information** | | | | |
| --- | --- | --- | --- | --- |
| **Characteristic features** | **EMCA** | **NCC** | **OHRM** | **TCGA** |
| Number of patients | 297 | 2120 | 1486 | 248 |
| Not hispanic or Latino | 290 (97.6%) | 1998 (94.2%) | 1445 (97.2%) | 193 (77.8%) |
| Other race/ethnicity or not reported | 7 (2.4%) | 113 (5.3%) | 30 (2.0%) | 55 (22.2%) |
| Average age (years) | 60.5 | 84.6 | 59.43 | 63.0 |
| Age range | 27-87 | 70-89 | 21-88 | 33-90 |
| Average BMI (kg/m^2^) | 38.0 | 27.1 | 29.42 | 33.66 |

| **Table S2: DiscovEHR and TCGA EMCA participant demographic information** | | |
| --- | --- | --- |
| **Tumor Type** | **DiscovEHR** | **TCGA** |
| Endometrioid Carcinoma (EEC) | 265 (89.2%) | 200 (80.6%) |
| Non-Endometrioid Carcinoma (NEEC) | 32 (10.8%) | 48 (19.4%) |
| Serous | 17 (5.7%) | 44 (17.7%) |
| Clear cell | 8 (2.7%) | NR |
| Undifferentiated (DiscvoEHR) or mixed (TCGA) | 2 (0.7%) | 4 (1.6%) |
| Carcinosarcoma | 5 (1.7%) | NR |

NR = Not reported

| **Table S3: Distribution of stages between races and studies as a percentage*** | | | |
| --- | --- | --- | --- |
| **Demographic** | **Stage 1**  **(localized)** | **Stage 2/3**  **(regional)** | **Stage 4**  **(distant)** |
| DiscovEHR EMCA | 80% | 18% | 2% |
| All TCGA | 71% | 23% | 5% |
| TCGA (White) | 77% | 20% | 3% |
| TCGA (Black) | 32% | 48% | 4% |
| All U.S. | 67% | 21% | 9% |
| U.S. (White) | 69% | 20% | 8% |
| U.S. (Black) | 53% | 25% | 16% |

*Percentages may not add to 100% due to unreported stages

**U.S. estimates from [1].

| **Table S4: Distribution of grades across studies for EMCA*** | | | |
| --- | --- | --- | --- |
| **Grade** | **DiscovEHR*** | **TCGA (N)** | **U.S. Estimates**** |
| Grade 1 | 50.8% (151) | 30.2% (75) | 72% |
| Grade 2 | 31.3% (93) | 30.6% (76) |  |
| Grade 3 | 7.7% (23) | 39.1% (97) | 27% |

*Percentage don’t add to 100% due to unreported grades for 30 samples.

**U.S. estimates based on [44].

| **Table S5: Total patient information for therapy and outcomes among DiscovEHR EMCA cohort** | |
| --- | --- |
| **Therapy** | |
| Surgery | 34 |
| Radiation only | 58 |
| Chemotherapy only | 27 |
| Chemotherapy and Radiation | 25 |
| No Further Treatment | 187 |
| **Recurrent Disease** | |
| Yes | 27 |
| No | 269 |
| Not reported | 1 |
| **Vital Status** | |
| Follow-up duration in years | 6.0 |
| Alive w/o disease | 257 |
| Alive with disease | 6 |
| Died of Disease (DOD) | 17 |
| Died- other causes | 17 |
